# Sustaining Continuous Engagement in Value Co-creation Among Individuals in Universities Using Online Platforms: Role of Knowledge Self-Efficacy, Commitment and Perceived Benefits

**DOI:** 10.3389/fpsyg.2021.637808

**Published:** 2021-02-12

**Authors:** Nabil Hasan Al-kumaim, Abdulsalam K. Alhazmi, T. Ramayah, Muhammad Salman Shabbir, Nadhmi A. Gazem

**Affiliations:** ^1^Faculty of Technology Management and Technopreneurship, Universiti Teknikal Malaysia Melaka (UTeM), Melaka, Malaysia; ^2^Faculty of Electronic and Distance Learning, University of Science and Technology, Aden, Yemen; ^3^School of Management, Universiti Sains Malaysia, Penang, Malaysia; ^4^Internet Innovation Research Center, Newhuadu Business School, Minjiang University, Fuzhou, China; ^5^Department of Management, Sunway University Business School (SUBS), Selangor, Malaysia; ^6^Faculty of Economics and Business, Universiti Malaysia Sarawak, Sarawak, Malaysia; ^7^College of Commerce and Business Administration, Dhofar university, Salalah, Oman; ^8^Management Information Systems, College of Business Administration-Yanbu, Taibah University, Madina, Saudi Arabia

**Keywords:** value co-creation, community engagement, online platform, commitment, self-efficacy, perceived benefits

## Abstract

Value Co-Creation (VCC) plays a major role in engaging knowledgeable individuals in a community *via* innovation, problem solving, and new service/product development. This study investigates the personal factors that influence individuals’ engagement in value co-creation in Higher Education Institutions (HEIs) through the use of online platforms. Some higher education institutions have successfully established or used appropriate online platforms, such as online forums, web applications, and mobile applications to engage their community in ideation or crowdsourcing as a part of the value co-creation process. On the other hand, some HEIs have failed to engage their community in value co-creation activities, and even if they managed to engage some individuals in value co-creation once, they failed to sustain these individuals’ engagement in value co-creation using online platforms. Using the Stimulus Organism Response (S-O-R) framework, this study examines the relationship between relevant personal factors (commitment and knowledge self-efficacy) and other motivational factors that provide perceived benefits with value co-creation engagement. Data was collected from 308 respondents at five Malaysian research universities. The software analysis tool Smart PLS is used for data analysis and validation. The results demonstrate that personal factors and perceived benefits as a motivational factor has a significant effect on individual engagement in value co-creation. However, the significance of these findings varies from one individual to another. The implications of these findings are discussed.

## Introduction

Customers are the source and focal element for co-created value (Vargo and Lusch, 2008). Customers’ personal, psychological, and competency perspectives have an impact on their level of online engagement in value co-creation. Some authors have emphasized the significant impact of monetary or materialistic reward systems on customer engagement with value co-creation (Füller et al., 2009). On the other hand, other scholars have addressed the specific influential aspects of obtaining benefits that generate various utilitarian, hedonic, or relational outcomes (Nambisan and Baron, 2009). However, these influential aspects might be more suitable for product co-production and co-designing rather than value co-creation in the service sector. Some of the abovementioned influential aspects may still motivate customers in HEIs to engage in value co-creation. While scholars have investigated value co-creation from many different perspectives, studies focusing on co-creation activities from a higher education viewpoint are limited (Sutarso et al., 2019). From the interactional perception, context and personality factors such as reputation, self-development, and altruism explain an individual’s motivation to contribute and share knowledge and experience (Oreg and Nov, 2008).

Firms and service organizations should pay more attention to the resources that customers can contribute, explore their potential to engage diverse individuals, and offer opportunities for more extensive value co-creation. Such engagement will facilitate intense individual participation and connection with an organization’s offerings or activities. As such, customer participation is viewed as a behavioral component in the interactive sphere. Moreover, customer involvement reflects the cognitive, affective, or motivational component of customer engagement in a service (Jaakkola and Alexander, 2014). On the other hand, the real challenge facing service providers is how to attract and motivate customers to engage in value co-creation processes and how to ensure their continual participation in value co-creation (Monavvarifad et al., 2019).

Previous studies have examined the role of social media, web applications, and online platforms in exploring customer motivations for participating in co-creation activities (Nambisan, 2002; Füller et al., 2009; Nambisan and Baron, 2009; Krishna et al., 2013). These studies have mostly focused on the customers’ perceived benefits as the main factor motivating customer online engagement behavior in value co-creation. However, few studies have investigated customer personal factors and their impact on customer online behavior (McElroy et al., 2007; Alan et al., 2017). To this end, the aim of this study is to empirically examine customer-personal factors that lead to value co-creation engagement, considering HEI technological capabilities that guarantee such useful interactions occur. The study extends the concept of influential value co-creation factors by adding two personal factors – participant commitment and knowledge self-efficacy – to the Stimulus Organism Response (S-O-R) framework. The study introduces a comprehensive model that theorizes how the impact of a number of personal variables can lead customers to engage in co-creation activities in HEIs.

Following this introduction, the rest of this article will be organized as follows: Part Two presents a comprehensive review of the literature, Part Three proposes the research model, and Part Four examines this study’s implications.

## Literature Review and Theoretical Background

### Co-creation Concept

Kambil et al. (1999) coined the term “co-creation” to refer to the shared creation of value between a company and its customers. In this process, the company proposes co-creation activities that give rise to a new dynamic between the company and the customer, as customers participate in the production process and the distribution of value. Co-creation stresses the integration of company or service provider resources with customers. However, for the concept of value co-creation to be meaningful and manageable, organizations need to know what they should manage. Customers can participate in every stage of the value chain, becoming partial “employees” of the organization (Kambil et al., 1999). Prahalad and Ramaswamy then used “co-creation” to refer to activities in which the consumer and company are both involved in the creation of value (Haro et al., 2014).

Good managers must learn new techniques to motivate customers to co-create value, as well as to find ways to successfully monitor and manage the process of co-creation in their organization.

### Value Co-creation in Higher Education Institutions

Innovative individuals in HEIs can be a valuable asset during the development process. The more visibly open providers are to receiving and implementing new ideas, the more opportunities they will receive to be in the front line of giving stakeholders the chance to meet the challenges of the future (Laine et al., 2008). The literature on Higher Education suggests that students can be viewed as “customers” and university lecturers/administrators can be seen as “service providers” (Redding, 2005).

However, other studies have indicated that universities are responding to two different types of clients. These can be classified as internal customers, such as *“students and employees, both academics and technical,”* and external customers, such as *“suppliers, financiers (creditors, NGOs, and funders), trade unions, parents, quality assurance bodies, trade associations, based on education process”* (Pereira and Da Silva, 2003). While many potential customers have long-term relationships with universities, many have also accumulated experience, knowledge, and skills by belonging to HEI professional bodies and standards associations. These resources represent a vital component of co-created value if they are integrated with an HEI’s resources. Customers in HEIs are therefore encouraged to introduce innovative and constructive ideas as well as to participate in generating value. Customers in HEIs should increasingly be motivated to express their engagement in value co-creation to better HEIs using their varying skills, experiences, and competencies (Hasan et al., 2015).

In addition, research on value co-creation engagement in the service sector in general is still at an immature stage, and there is a need for further investigation into how this engagement can be sustained through an effective technology platform, especially amongst HEIs (Fadeeva and Mochizuki, 2010; Waas et al., 2010; Monavvarifad et al., 2019). While customer participation in the service sector is not novel, what is new is the acknowledgment that HEIs only provide partial input into a customer’s experience (McColl-Kennedy et al., 2009). It is therefore necessary to co-opt and empower HEI customers to take a role in value co-creation in the education sector (Vargo and Lusch, 2008). Co-creation through HEIs is a vital activity that enables sustainable development, social innovation, and community transformation, thus driving HEIs to become world leaders in developing successful international partnerships with businesses and communities (Laine et al., 2008; Kumari et al., 2020).

### Related Theoretical Work

Prior studies on value co-creation have examined the determinants of customer participation, with a focus on the new product development stage (Füller et al., 2009; Hoyer et al., 2010) and the product support stage (Nambisan and Nambisan, 2008; Nambisan and Baron, 2009). For instance, Füller et al. (2009) investigated the determinants of consumer intentions to participate in co-creation activities during new product development from a consumer empowerment perspective. In another study, Füller (2006) examined six motivational factors that impact customer engagement in value co-creation through virtual development activities in the online community, which were monetary rewards, showing ideas, gaining knowledge, intrinsic interest, dissatisfaction, and curiosity. Hoyer et al. (2010) presented a conceptual framework that examined the experiences and consequences of co-creation throughout new product development processes with respect to consumer co-creation. Meanwhile, Nambisan and Baron (2009) examined the experiences of customer participation in product support from an integrative perspective that incorporated interaction-based customer benefits and affective evaluation using the Use and Gratification (U and G) framework (Katz and Blumler, 1974). The U and G framework identifies four comprehensive benefit types: cognitive benefits, social integrative benefits, personal integrative benefits, and hedonic benefits that individuals can derive from media usage. A number of relevant studies have examined why customers participate in co-creation activities on firm-initiated online platform sites. They have found that customer participation in value co-creation was determined by customer learning values, social integrative values, and hedonic experiences, which were influenced by perceived task-relevant and affection-relevant causes (Zhang et al., 2015; Roy et al., 2019).

### S–O–R Framework

The current study’s research model was developed based on the S–O–R framework (Mehrabian and Russell, 1974). This outlines how different Stimuli (S) influence individual internal Organisms (O) to derive behavioral Responses (R). S-O-R theory has been used in many studies to investigate the factors that influence customer behaviors toward value co-creation in online contexts. These behaviors include participation, intention for future participation, and engagement in VCC activities through online platforms (Chuang and Lin, 2014; Chuang and Chen, 2015; Zhang et al., 2015; Zhang and Benyoucef, 2016).

This study uses the S–O–R framework as a theoretical base for two reasons. First, the S–O–R framework has been used in previous studies on online customer behavior, and their findings have affirmed that S-O-R is an appropriate framework for explaining customer reactions and behavioral responses to the different types of stimuli. Second, by investigating the roles of certain personal factors and perceived benefits in influencing value co-creation engagement behaviors using online platforms, the S–O–R framework provides an acceptable structured approach for examining the effects of personal factors (stimuli) on customer’s internal reactions to perceived benefits (internal organisms) and their engagement in co-creation activities (external response).

## Research Model and Hypothesis Development

### Individual Commitment in HEIs Communities and Perceived Benefits

The term “commitment” has been defined as an individual’s pride in belonging, their concern for long-term success, and a desire as a customer to contribute toward the betterment of an organization (Morgan and Hunt, 1994). Previous empirical studies conducted in online settings has shown that individuals engage in value co-creation activities and share knowledge because they expect to obtain learning benefits, such as enhanced knowledge (Greve et al., 2016). Commitment can be the result of a cognitive calculation or an emotional attachment (Geyskens et al., 1996; Wetzels et al., 1998). This study therefore considers HEIs to be part of the service sector, which makes affective commitment underlie loyalty decisions since commitment is founded upon cognitive calculation. Based on the literature review, this study created the following hypothesis:

H1a: Commitment is positively associated with learning benefits in the context of value co-creation engagement in HEIs.

In addition, an individual’s willingness to share their own ideas and knowledge *via* online co-creation platforms is motivated by intangible rewards such as improved reputation, image, or status (Jahani et al., 2013; Šajeva, 2014). However, some research has found that reputation did not have a significant impact on knowledge sharing in the academic environment (Mallasi and Ainin, 2015). In this study, we modified a definition of reputation used in previous studies to fit our study context, as there is a perception that reputation increases when knowledge is contributed during value co-creation on online platforms (Kankanhalli et al., 2005). This study thus proposes the following hypothesis:

H1b: Commitment is positively associated with reputation improvements in the context of value co-creation engagement in HEIs.

“Intrinsic benefits” are obtained when individuals derive enjoyment and pleasure from helping others without expecting something in return (Krebs, 1975; Ryan and Deci, 2000). Prior studies on online platforms have found that knowledge contributors gain satisfaction from pursuing altruistic behavior and are willing to engage in intellectual exposure and problem-solving to contribute knowledge because they are intrinsically motivated to face challenges, to pursue satisfaction, or enjoy helping others (Wasko and Faraj, 2000, 2005). As an individual’s willingness to share knowledge has been proven to have a significant relationship with intrinsic benefits in the context of knowledge sharing, it is assumed that the same significant relationship will exist in the context of value co-creation online engagement. Therefore:

*H1c: Commitment is positively associated with intrinsic benefits in the context of value co-creation engagement in HEIs*.

Some researchers have defined extrinsic rewards as benefits, payments, or career prospects that are given in return for the participation of some individuals in value co-creation (Füller, 2010). The term “extrinsic benefits” denotes the tangible benefits offered by organizations to individuals or employees (Newman and Sheikh, 2012). Organizations can offer different forms of financial rewards to motivate individuals to share knowledge and add value to their organization, such as an increase in salary, job security, bonuses, or promotions (Kankanhalli et al., 2005; He and Wei, 2009). It is therefore expected that individuals who are satisfied with their extrinsic benefits will have a greater affective commitment toward the organization in return (Newman and Sheikh, 2012). Therefore:

*H1d: Commitment is positively associated with extrinsic benefits in the context of value co-creation engagement in HEIs*.

### The Effect of Knowledge Self-Efficacy on Perceived Benefits

A belief in self-efficacy is necessary for an individual to be considered a value co-creator or co-producer (Jacob and Rettinger, 2011). In contrast, when a person believes that they are incapable of performing a particular task, they will not engage in that behavior, even if they acknowledge that it is a better alternative (Meuter et al., 2005). Knowledge self-efficacy typically manifests when a person believes that their knowledge can help solve job-related problems (Constant et al., 1996) or make a difference in their organization (Wasko and Faraj, 2000). Knowledge self-efficacy is the confidence in one’s ability to provide knowledge that is valuable to an organization via online platforms (Constant et al., 1996; Kalman, 1999; Kankanhalli et al., 2005). Knowledge self-efficacy plays an important role in acquiring the desired knowledge, making it an important factor when determining the effectiveness of a new learning strategy (Kuznar, 2009). Therefore:

H2a: Knowledge self-efficacy is positively associated with learning benefits in the context of value co-creation engagement in HEIs.

Social benefits such as status, image, and respect cause behaviors to increase in intensity (Hoisl et al., 2007). Previous investigations into methods of triggering creativity and ideas found that social benefits such as reputation and image positively affected an individual’s self-esteem, which is part of self-efficacy (Hung et al., 2011). Additionally, other scholars have suggested that individuals who share their experience and knowledge with others will gain benefits such as enhanced self-image, reputation, and status in their social circle (Wasko and Faraj, 2005; Hsu et al., 2007). Therefore:

H2b: Knowledge self-efficacy is positively associated with image benefits in the context of value co-creation engagement in HEIs.

Previous studies on knowledge contributions to electronic repositories found that knowledge self-efficacy and enjoyment in helping others were intrinsic benefits that had significant and positive relationships with using online platforms to share knowledge (Kankanhalli et al., 2005). To find the relationship between Knowledge Self-Efficacy and enjoyment in helping others as an intrinsic benefit, this study thus proposes:

*H2c: Knowledge self-efficacy is positively associated with intrinsic benefits in the context of value co-creation engagement in HEIs*.

Scholars studying behavioral intention formation in knowledge sharing have argued that extrinsic rewards do not represent a primary motivator within knowledge-sharing initiatives. However, there is a consensus on the importance of enhancing individual self-efficacy to engage individuals in knowledge-sharing behaviors (Bock et al., 2005). To examine the relationship between Knowledge Self-Efficacy and extrinsic benefits, this study therefore proposes:

*H2d: Knowledge Self-Efficacy is positively associated with extrinsic benefits in the context of value co-creation engagement in HEIs*.

### The Effect of Learning Benefits

In the process of co-creating value through online platforms, some customers are motivated by gaining learning benefits and acquiring knowledge about a technology-based product or developing new services (Nambisan and Baron, 2009). Etgar (2008) highlights the importance of considering the motivations of the customer to participate in co-creation (Etgar, 2008). Similarly, Payne et al. (2008) emphasized that customer learning is one of the central parts of co-creation (Payne et al., 2008). Etgar (2008) points out that customer learning is a source of motivation to participate in co-creation. Moreover, customers might decide to participate because they enjoy the process (Etgar, 2008). Based on the literature, we hypothesize:

H3: Learning benefits will have a positive influence on value co-creation engagement by HEI communities.

### The Effect of Social Benefits (Image)

Some participants in value co-creation may receive social benefits, such as titles or other forms of recognition, that a firm might bestow on particularly valuable contributors. The social benefits of co-creation include increased status, social esteem, “good citizenship,” and the strengthening of ties with other relevant actors (Nambisan and Baron, 2009). When organizations apply value co-creation activities, such as the generation of ideas through crowdsourcing, it is also important to highlight that the reward is always given by the initiator of the crowdsourcing initiative (provider). While there can be a secondary reward, such as social recognition from other crowdsourcing participants, these rewards are not the focus and do not need to be presented. It can therefore be concluded that the user will obtain satisfaction of a given necessity, whether it be economic, social recognition, self-esteem, or the development of individual skills (Estellés-Arolas and González-Ladrón-de-Guevara, 2012). Based on the literature, this study proposes the following hypothesis:

H4: Social benefits will have a positive influence on value co-creation engagement by HEI communities.

### Intrinsic Benefits

Past studies on motivations for value co-creation in the context of open innovation projects have shown that there are multiple reasons that customers engage in value co-creation activities. Some of these are purely intrinsic motives, such as fun, kinship, and altruism (Füller, 2010). By co-creating value, contributors feel satisfied because they gain a strong feeling of achievement. The sense of creating something or contributing to something important to your peers or society has a bigger impact than just deriving value from the provider (Krishna et al., 2013). There are thus some participants in value co-creation who are influenced by intrinsic motivations (San Cornelio and Gomez Cruz, 2014), personal drives as altruism or self-reward, or the pleasure of taking part in an activity (Janzik and Herstatt, 2008). It is interesting to note that a number of previous studies have suggested that intrinsic benefits cause value co-creators to become involved in development activities (i.e., attitudes, intentions, and participation) (Maurer et al., 2003). Based on the literature, this study proposes the following hypothesis:

H5: Intrinsic Benefits will have a positive influence on value co-creation engagement by HEI communities.

### Extrinsic Benefits

Some researchers have defined purely extrinsic rewards or benefits as the granting of payments or career prospects in return for the participation of individuals in value co-creation (Füller, 2010). Some individuals who participate in value co-creation are “*motivated by financial rewards, either in a direct way in the form of financial prizes or revenues sharing from the firm that engages in co-creation activates, or indirectly, through the intellectual property that they might receive, or through the recognition that they might receive from being engaged in co-creation competitions*” (Hoyer et al., 2010). Nevertheless, many others are not purely motivated by money: they choose to “free reveal” ideas and freely share effort in the post-ideation stages of co-creation (Franke et al., 2005). Based on the literature, this study proposes the following hypothesis:

H6: Extrinsic Benefits will have a positive influence on value co-creation engagement by HEIs.

### Mediation Effect

The mediation effect occurs when a third variable (the mediator variable) intervenes in the relationship between the independent and dependent variables (Preacher and Hayes, 2008). Mediation is also known as the “indirect effect,” which requires theoretical or conceptual support to explore meaningful mediation effects (Hayes and Preacher, 2011; Hair et al., 2016), as the main objective in mediation analysis is explaining how a given effect occurs (Henseler et al., 2016). According to Ramayah et al. (2018), when support exists for the mediating relationship, mediation can be a useful tool for statistical analysis. This study thus expects to have multiple mediation effects, as follows:

H7: The effect of Commitment on Engagement will be mediated by Learning Benefits

H8: The effect of Knowledge self-efficacy on Engagement will be mediated by Learning Benefits

H9: The effect of Commitment on Engagement will be mediated by Image

H10: The effect of Knowledge self-efficacy on Engagement will be mediated by Image

H11: The effect of Commitment on Engagement will be mediated by Intrinsic Benefits

H12: The effect of Knowledge self-efficacy on Engagement will be mediated by Intrinsic Benefits

H13: The effect of Commitment on Engagement will be mediated by Extrinsic Benefits

H14: The effect of Knowledge self-efficacy on Engagement will be mediated by Extrinsic Benefits.

## Research Methodology and Data Collection

The model used in this study is complex, with two independent variables, four mediators, and one multi-dimensional dependent variable. This study therefore used Partial Least Square-Structural Equation Modeling (PLS-SEM) using SmartPLS v. 3.2.8 (Ringle et al., 2015) as a statistical tool to examine the measurement and structural model. This approach is particularly suited to this paper as it can accommodate smaller sample sizes without normality assumptions, which is required as survey research is not normally distributed (Chin et al., 2003). This study followed the guidelines of Anderson and Gerbing (1988) and tested the measurement model using a two-step approach, which was followed by the assessment of the structural model (Anderson and Gerbing, 1988).

The two personal independent variables comprised a knowledge self-efficacy factor represented by items adopted from Kankanhalli et al. (2005), and a commitment factor with four items adopted from Bozeman and Perrewé (2001). The four perceived benefit variables included 15 items: (four items for learning benefits, five items for social benefits (image), three items for intrinsic benefits, and four items for extrinsic benefits). The response scale used a 5-point Likert scale with 1 = strongly disagree to 5 = strongly agree. For the dependent variable (value co-creation engagement), which is a multi-dimensional construct, the “represented in web engagement” variable with four items was adopted from Webster and Ahuja (2006) while the “interactional engagement” variable with four items was adopted from So et al. (2014). The items for all of these constructs listed as Supplementary Materials attached with this research paper see Supplementary Table 6.

The population of this study included students and staff from five public research universities in Malaysia. These five public research universities were chosen because they had already implemented value co-creation activities utilizing specific online platforms. Moreover, potential respondents should have experience with using university online platforms to participate in value co-creation related activities.

According to Anderson and Gerbing (1984), the minimum sample size to make appropriate estimates is 100–150 subjects. However, other authors recommend a minimum sample size of 200 respondents (Hoelter, 1983). To ensure the adequacy of the sample size, G^∗^power3 software was therefore used (Faul et al., 2007). The setting proposed by Dattalo (2008) was used (α = 0.05, β = 0.8, and effect size *f*^2^ = 0.15). Supplementary Figure 2 shows that at error probability of 0.05, and confidence level 80%, the minimum required sample size for this study is 98. This result indicates that the sample of 308 collected surveys used in this study is adequate.

The survey was conducted using self-administered questionnaires that were distributed to the target group, members of which were selected using a purposive sampling technique. The survey for this study was carefully designed and validated in several stages, including face validity, content validity, and validity pretests. Both online and printed questionnaires were distributed to targeted universities, and after incomplete and unreturned questionnaires were excluded, only 308 questionnaires were valid for analysis. The respondent profile was determined using the frequencies and percentages shown in Supplementary Table 1. The majority of participants were female (68%), while 32% were male. The respondents were relatively young, being mainly 20 to 35 years old (67%), while 33% were older. Supplementary Table 1 summarizes the respondent’s demographic information.

## Results

### Measurement Model Assessment

To assess the measurement items and constructs, this study tested both convergent and discriminant validity. The tests for reliability and convergent validity are presented in Supplementary Table 2. This study used composite reliability to assess reliability and values more than 0.7, which is considered sufficient (Hair et al., 2016; Memon et al., 2017). Convergent validity, which assesses the degree to which items are related to the construct as theoretically conceptualized, were checked using the item loadings and Average Variance Extracted (AVE) for each construct (Hair et al., 2016; Memon et al., 2017). All item loadings exceeded 0.7, and for AVE all constructs exceeded the threshold value of 0.50 (Fornell and Larcker, 1981), indicating adequate convergent validity in the measurement model. VCC Engagement is a second-order factor with two lower-order dimensions (IEG and WEG) that had a loading of 0.818 and 0.900, an AVE of 0.818, and a Composite reliability of 0.900, which also passes the convergent validity test.

The term “discriminant validity” refers to the degree to which constructs hold discrimination to each other. This study tested discriminant validity using the heterotrait-monotrait ratio of correlations (Henseler et al., 2015), as shown in Supplementary Table 3. If a HTMT value is greater than 0.85 (Kline, 2015), then there is a discriminant validity problem, whereas a value smaller than 0.85 signals discriminant validity is good. As all HTMT values were less than 0.85 (Kline, 2015), as shown in Supplementary Table 3, good discriminant validity was ascertained. Both assessments show that the measurement items were valid and reliable, thus allowing for hypothesis testing.

### Structural Model Assessment

This study tested the structural model by assessing the explained variance (R^2^), path coefficients, and corresponding *t*-values. This study used a bootstrapping procedure with 5,000 resamples (Hair et al., 2016; Memon et al., 2017) to derive a valid standard error for *t*-value calculation. This study first analyzed the effect of personal factors on perceived benefits, followed by the effect of perceived benefits on VCC engagement and indirect effects. Commitment and knowledge self-efficacy together explained 17.8% (Extrinsic Benefits), 37.4% (Social Benefits), 44.7% (Intrinsic Benefits), and 43.1% (Learning Benefits) of variance in VCC Engagement. All four perceived benefits explained 50.6% of the variance in VCC Engagement.

For the direct relationships (see Supplementary Table 4), the study’s first (H1a,b,c,d) hypothesis states the commitment has a positive relationship with Extrinsic Benefits, Image, Intrinsic Benefits, and Learning Benefits. The results indicate path coefficients are 0.317, 0.104, 0.355, and 0.044, with *t* values of 4.989, 1.648, 5.416, and 0.886 for one tiled test. This indicates that hypotheses Ha,b, and c are accepted while hypothesis Hd is rejected.

Hypothesis H2 indicates that knowledge self-efficacy has a positive relationship with Extrinsic Benefits, Image, Intrinsic Benefits, and Learning Benefits. The results indicate path coefficients are 0.405, 0.559, 0.382, 0.393 with *t* values of 7.153, 8.433, 5.773, and 4.872 for one tiled test. The results indicate that knowledge self-efficacy has a positive relationship with Extrinsic Benefits, Image, Intrinsic Benefits, and Learning Benefits. Similarly, hypotheses H3, H4, H5, and H6 indicate that Extrinsic Benefits, Image, Intrinsic Benefits, and Learning Benefits have a positive relationship with engagement. The results indicate that the path coefficients are 0.131, 0.145, 0.140, and 0.138, respectively, with *t* values of 3.505, 3.658, 4.479, and 2.849. This indicates a significant positive relationship. Hypotheses H3, H4, H5, and H6 are thus accepted.

For testing the mediation effect in PLS-SEM, the application of bootstrapping has been recognized by researchers as the more rigorous and powerful application available for inference statistics (Hayes, 2009; Henseler et al., 2009; Zhao et al., 2010; Hair et al., 2013). In the present research, the bootstrapping technique was used with 500 sub-samples to generate approximate *t*-values for significance testing of all the path coefficients. Supplementary Table 5 below presents the t-statistics values.

Hypotheses H7, H9, and H11 to H13 state the indirect effect of commitment on engagement through Extrinsic Benefits, Image, Intrinsic Benefits, and Learning Benefits. The path coefficients of indirect paths were reported to be 0.078, 0.023, 0.097, and 0.006, respectively, with *t* values of 2.673, 0.175, 3.234, and 0.572. This indicates hypotheses H7 and H11 were accepted and hypotheses H9 and H13 were rejected. Similarly, Hypotheses H8, H10, and H12 to H14 refer to the indirect effect of self-efficacy on engagement through Extrinsic Benefits, Image, Intrinsic Benefits, and Learning Benefits. The path coefficients of indirect paths were reported to be 0.100, 0.122, 0.105, and 0.056, respectively, with *t* values of 2.942, 3.022, 3.450, and 2.042. This indicates hypotheses H8, H10, H12, and H14 were accepted. Supplementary Table 5 shows the results of indirect effects.

## Discussion and Implications

### Discussion

After examining the validity and reliability of the constructs, this study tested the proposed hypotheses using Smart PLS. The results demonstrated that all direct relationships between the two personal factors (individual commitment and knowledge self-efficacy) had a significant effect on all four perceived benefits, as H1a, H2a, H1b, H2b, H1c, H2c, H2d, H3, H4, H5, and H6 were supported while H1d was not supported. Hypothesis H1d tested the relationship between individual commitment and extrinsic benefits, and it not being supported shows that individual commitment to participate in value co-creation activities was not significantly influenced by extrinsic benefits.

Extrinsic benefits in the context of this study were defined as an individuals’ belief that monetary or tangible incentives will be given in return for ideas and knowledge-sharing as part of value co-creation activities. The results of this study appear to reject the hypothesis that an individual’s commitment has a positive relation with extrinsic benefits when engaging in value co-creation. This is inconsistent with the findings of other researchers who found a significant relationship between extrinsic rewards and individual attitudes toward knowledge-sharing (O’Reilly and Pondy, 1979; Quinn et al., 1998; Liebowitz, 2002). This inconsistency might have arisen for several reasons. First, the context of this study is academic, and for academic staff and students, monetary and tangible rewards may not significantly contribute to the formation of commitments to share knowledge and experience in value co-creation activities (Welschen, 2014).

Participants in value co-creation at universities may therefore place greater value on intangible rewards and benefits than tangible benefits (Gustad, 1960). Moreover, this study was conducted in 5 public research Universities in Malaysia, where the participants were predominantly Muslims. It should therefore be taken into account that some participants might consider experience-sharing for value creation to be encouraged according to Islamic beliefs and teaching as a spiritually rewarded behavior regardless of any tangible rewards offered in return (Jolaee et al., 2014). Participants may thus share their knowledge and experience without consideration for monetary rewards. The results of this study therefore confirm that there is a significant relationship between individual commitment and knowledge self-efficacy with the perceived benefits from engaging participants in HEIs in online value co-creation activities. The results of this study demonstrate a significant relationship between perceived benefits and value co-creation engagement, as shown in Supplementary Table 3.

On the other hand, as illustrated in Supplementary Table 5, the results show that six out of eight indirect relationships were significant (H7, H8, H10, H11, H12, and H14) while the other two indirect relationships (H9 and H13) were insignificant. It is thus found that an individual’s knowledge self-efficacy has a significant indirect relationship on an individual’s engagement in value co-creation through all of the four perceived benefits. This result is consistent with the previous value co-creation literature that emphasized the importance of a participant’s confidence, along with their ability to provide knowledge that is valuable to the organization via online platforms (Constant et al., 1996; Kalman, 1999; Kankanhalli et al., 2005). According to Vargo and Lusch (2004), knowledge and skills are the most important types of resource that enable co-creation of value to occur according to the service-dominant logic (S-D) concept (Vargo and Lusch, 2004; Campbell et al., 2013).

In addition, individual commitment has a significant and indirect relationship on individual engagement in value co-creation through learning benefits and intrinsic benefits. This is consistent with the value co-creation literature in that a participant’s sense of commitment positively influences customer engagement in value co-creation activities through online HEI platforms (Brodie et al., 2013; Bugshan, 2015). A sense of commitment thus strongly motivates staff and students in universities to contribute ideas and engage in value co-creation activities through online university platforms.

Only two relationships, H9 and H13, were found to be insignificant as to extrinsic benefits and image representing social benefits. These findings are logically and analytically acceptable for an indirect relationship, because they almost reflect the significance of the direct relationship seen in Supplementary Table 3 in relation to the commitment and extrinsic benefits. Regarding the relationship between commitment, image, and engagement, the COM → IMG → Engagement results revealed an insignificant relation. This might be because participants dealt with image and reputation benefits in the same way they evaluated and dealt with extrinsic benefits.

### Theoretical and Practical Implications

This study contributes to the value co-creation literature by demonstrating that personal factors (individual’s commitment, knowledge self-efficacy, and perceived benefits) have a great influence on customer engagement in value co-creation in higher education institutions through the use of online platforms. This result contributes to the value co-creation literature on the importance of the relationship between personal factors and value co-creation engagement. In addition, the study also demonstrated that knowledge self-efficacy and commitment plays a significant role in individual online engagement in value co-creation activities. High levels of commitment, knowledge self-efficacy and perceived benefits lead to high levels of engagement with online value co-creation activities.

The study also has some practical implications. First, although previous studies found that perceived benefits motivate potential participation in value co-creation (McElroy et al., 2007; Alan et al., 2017), the findings of this study imply that HEIs should pay more attention to fostering and engaging individuals with knowledge self-efficacy and commitment as they are very significant stimuli for engaging in value co-creation. This can be implemented *via* workshop training, seminars, and gatherings within the HEI community designed to encourage individuals to practice affective commitment and gain knowledge self-efficacy through continual participation in value co-creation activities.

Second, to maximize and maintain high levels of value co-creation engagement behavior among HEI communities using online platforms, great consideration should be given to both how attractive a platform is and also how organizations can maximize perceived benefits and encourage individuals to have high commitment and self-efficacy.

### Directions for Further Studies

While this study contributes to the literature in both theory and practice, it still has a number of limitations that need to be considered. This study was conducted in a Malaysian context, meaning cultural and environmental differences must be considered when generalizing its findings. Moreover, survey data was collected from only five public universities. Further research could expand the survey to cover students from different public and private universities.

The units of analysis in this study included only a limited number of HEI internal customers as targeted respondents, such as students, academic staff, non-academic staff, and administrators. However, external stakeholders, which might include parents, related government agencies, industries, NGOs and other associated educational entities, were not included as target respondents in this study. Since this study mainly introduced personal factors that cause HEIs communities to engage in value co-creation through the use of online platforms, future studies might include other relevant elements such as online platform characteristics or other personal and organizational factors.

## Conclusion

This study suggests a theoretical model of the influence of personal factors mediated by four variables of perceived benefits on value co-creation engagement behavior in the HEI service sector. Moreover, this study explored and incorporated significant variables (commitment, knowledge self-efficacy, and perceived benefits) using the S-O-R framework to test how these variables cause individuals in the HEI community to engage in value co-creation activities. The results revealed that commitment, knowledge self-efficacy, and the mediative impact of perceived benefits had a positive effect on continuous customer engagement in value co-creation in higher education institutions with online platforms.

## Data Availability Statement

The original contributions presented in the study are included in the article/Supplementary Material, further inquiries can be directed to the corresponding author.

## Author Contributions

NA-K and TR provided data collection and initial analysis. NG, MS, and AA worked on the results, conceptualization, and methodology. AA, MS, and NA-K provided writing, review, and editing. All authors contributed to this study, read, and agreed to the published version of the manuscript.

## Conflict of Interest

The authors declare that the research was conducted in the absence of any commercial or financial relationships that could be construed as a potential conflict of interest.
