# Supplementary material for: Sustaining Continuous Engagement in Value Co-creation Among Individuals in Universities Using Online Platforms: Role of Knowledge Self-Efficacy, Commitment and Perceived Benefits
Source: Front Psychol. 2021 Feb 12;12:637808. doi: 10.3389/fpsyg.2021.637808 (PMC7907507; doi:10.3389/fpsyg.2021.637808)
Supplement: Supplementary file 1 [file Table_1.DOCX]

Supplementary Material

Sustaining Continues Engagement in Value Co-creation among Individuals in Universities Using Online Platforms: Role of Knowledge Self-Efficacy, Commitment and Perceived Benefits

# Supplementary Figures and Tables

Here is the relevant supplementary materials for the abovementioned study.

## Supplementary Figures


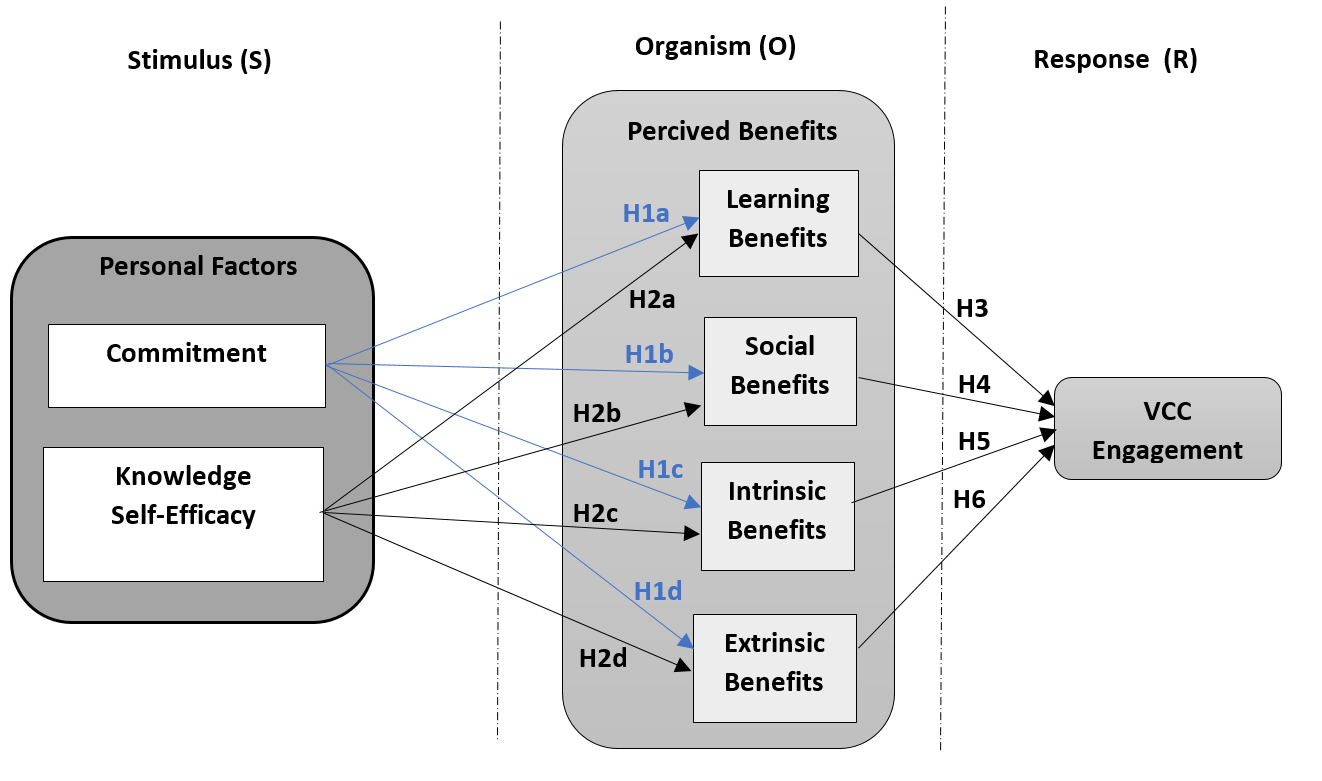


**Figure 1.** Research Model Based on S-O-R Framework


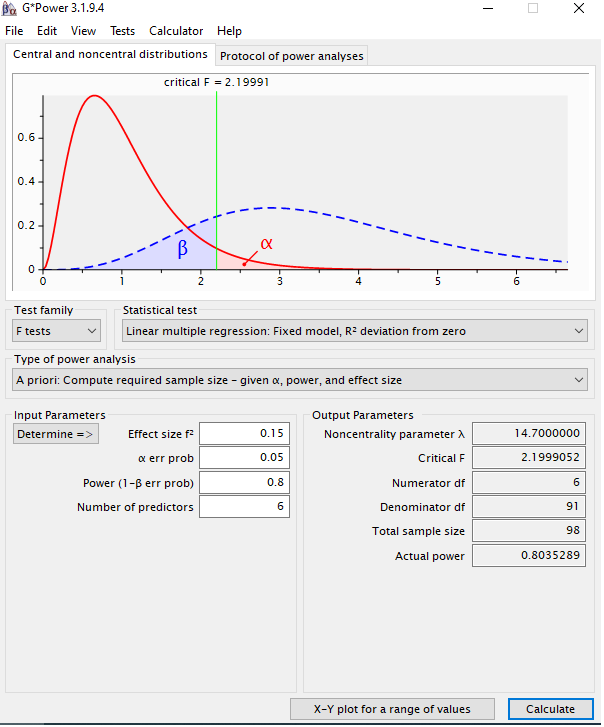


**Figure 2.** G*power results for required sample size.

## Supplementary Tables

**Table 1.** Respondent Demographic Profiles

| Respondent Information | Data category | Frequency (N=308) | Percentage  (100%) |
| --- | --- | --- | --- |
| Gender | Male | 98 | 32 |
|  | Female | 210 | 68 |
| Nationality | Malaysian | 299 | 97 |
|  | International | 9 | 3 |
| Level of Education | SPM | 34 | 11.1 |
|  | STPM | 11 | 3.7 |
|  | Diploma | 48 | 15.5 |
|  | Bachelor | 85 | 27.2 |
|  | Master | 100 | 32.4 |
|  | Doctorate | 22 | 7.4 |
|  | Post Doctorate | 8 | 2.8 |
| Designation | Staff | 180 | 58.8 |
|  | Students | 125 | 40.2 |
|  | Both | 3 | 0.8 |
| Participating in VCC activities in the university | Via online platform | 31 | 10.2 |
|  | Both | 275 | 89.2 |
|  | Non-online platforms | 2 | 0.6 |

**Table 2.**  Measurement Model

| Construct | Item | Loadings | CR | AVE |
| --- | --- | --- | --- | --- |
| Commitment | COM1 | 0.880 | 0.940 | 0.797 |
|  | COM2 | 0.874 |  |  |
|  | COM3 | 0.919 |  |  |
|  | COM4 | 0.899 |  |  |
| Extrinsic Benefit | EXB1 | 0.883 | 0.920 | 0.793 |
|  | EXB2 | 0.916 |  |  |
|  | EXB3 | 0.872 |  |  |
| Interaction Engagement | IEG1 | 0.900 | 0.946 | 0.814 |
|  | IEG2 | 0.924 |  |  |
|  | IEG3 | 0.888 |  |  |
|  | IEG4 | 0.897 |  |  |
| Social Benefits | IMG1 | 0.847 | 0.937 | 0.748 |
|  | IMG2 | 0.896 |  |  |
|  | IMG3 | 0.893 |  |  |
|  | IMG4 | 0.871 |  |  |
|  | IMG5 | 0.814 |  |  |
| Intrinsic Benefits | INB1 | 0.921 | 0.954 | 0.839 |
|  | INB2 | 0.927 |  |  |
|  | INB3 | 0.925 |  |  |
|  | INB4 | 0.892 |  |  |
| Knowledge Self-Efficacy | KSE1 | 0.824 | 0.878 | 0.647 |
|  | KSE2 | 0.863 |  |  |
|  | KSE3 | 0.881 |  |  |
|  | KSE4 | 0.622 |  |  |
| Learning Benefits | LRB1 | 0.906 | 0.932 | 0.820 |
|  | LRB2 | 0.885 |  |  |
|  | LRB3 | 0.926 |  |  |
| Web Engagement | WEG1 | 0.896 | 0.955 | 0.842 |
|  | WEG2 | 0.940 |  |  |
|  | WEG3 | 0.916 |  |  |
|  | WEG4 | 0.918 |  |  |

**Table 3.** Discriminant Validity

| Construct | 1 | 2 | 3 | 4 | 5 | 6 | 7 |
| --- | --- | --- | --- | --- | --- | --- | --- |
| 1. Commitment |  |  |  |  |  |  |  |
| 2. Extrinsic Benefit | 0.332 |  |  |  |  |  |  |
| 3. Engagement | 0.626 | 0.531 |  |  |  |  |  |
| 4. Social Benefits | 0.507 | 0.685 | 0.645 |  |  |  |  |
| 5. Intrinsic Benefits | 0.648 | 0.359 | 0.618 | 0.551 |  |  |  |
| 6. Self-Efficacy | 0.739 | 0.514 | 0.758 | 0.737 | 0.691 |  |  |
| 7. Learning Benefits | 0.639 | 0.531 | 0.667 | 0.675 | 0.654 | 0.711 |  |

**Table 4.** Hypothesis Testing (Direct Relationships)

| Hypothesis | Relationship | Std Beta | Std Error | t-value | p-value | f^2^ | Decision |
| --- | --- | --- | --- | --- | --- | --- | --- |
| H1a | **COM 🡪 LRB** | **0.317** | **0.064** | **4.989** | **0.000** | **0.103** | Significant |
| H2a | **KSE 🡪 LRB** | **0.405** | **0.057** | **7.153** | **0.000** | **0.168** | Significant |
| H1b | **COM 🡪 IMG** | **0.104** | **0.063** | **1.648** | **0.050** | **0.020** | Significant |
| H2b | **KSE 🡪 IMG** | **0.559** | **0.066** | **8.433** | **0.000** | **0.303** | Significant |
| H1c | **COM 🡪 INB** | **0.355** | **0.065** | **5.416** | **0.000** | **0.133** | Significant |
| H2c | **KSE 🡪 INB** | **0.382** | **0.066** | **5.773** | **0.000** | **0.154** | Significant |
| H1d | COM 🡪 EXB | 0.044 | 0.050 | 0.886 | 0.188 | 0.001 | Not significant |
| H2d | **KSE 🡪 EXB** | **0.393** | **0.081** | **4.872** | **0.000** | **0.110** | Significant |
| H3 | **LRB 🡪 Engagement** | **0.246** | **0.070** | **3.505** | **0.000** | **0.062** | Significant |
| H4 | **IMG 🡪 Engagement** | **0.218** | **0.060** | **3.658** | **0.000** | **0.045** | Significant |
| H5 | **INB 🡪 Engagement** | **0.274** | **0.061** | **4.479** | **0.000** | **0.092** | Significant |
| H6 | **EXB 🡪 Engagement** | **0.141** | **0.050** | **2.849** | **0.002** | **0.025** | Significant |

Note: Bolded items indicate statistically significant results (one-tail)

COM = Commitment, KSE= Knowledge Self-Efficacy, EXB= Extrinsic Benefits, IMG= Image, INB = Intrinsic Benefits, LRB= Learning Benefits.

**Table 5.** *Indirect Relationships*

| Hypothesis | *Indirect Relationships* | Std Beta | Std Error | t-value | p-value | Level of significant |
| --- | --- | --- | --- | --- | --- | --- |
| H7 | **COM 🡪 LRB 🡪 Engagement** | **0.078** | **0.029** | **2.673** | **0.008** | Significant |
| H8 | **KSE 🡪 LRB 🡪 Engagement** | **0.100** | **0.034** | **2.942** | **0.003** | Significant |
| H9 | COM 🡪IMG 🡪 Engagement | 0.023 | 0.017 | 1.357 | 0.175 | Insignificant |
| H10 | **KSE 🡪 IMG 🡪 Engagement** | **0.122** | **0.040** | **3.022** | **0.003** | Significant |
| H11 | **COM 🡪 INB 🡪 Engagement** | **0.097** | **0.030** | **3.234** | **0.001** | Significant |
| H12 | **KSE 🡪 INB 🡪 Engagement** | **0.105** | **0.030** | **3.450** | **0.001** | Significant |
| H13 | COM 🡪 EXB 🡪 Engagement | 0.006 | 0.011 | 0.572 | 0.568 | Insignificant |
| H14 | **KSE 🡪 EXB 🡪 Engagement** | **0.056** | **0.027** | **2.042** | **0.042** | Significant |

Note: Bolded items indicate statistically significant results (two-tail)

COM = Commitment, KSE= Knowledge Self-Efficacy, EXB= Extrinsic Benefits, IMG= Image, INB = Intrinsic Benefits, LRB= Learning Benefits.

**Table 6.** Items related to personal factors

| Factor | Item Code | Adopted items | Source of Items |
| --- | --- | --- | --- |
|  |  |  |  |
| Knowledge  self-efficacy | KSE1 | I have confidence in my ability to provide knowledge that others in my institution consider valuable. | (Kankanhalli *et al.*, 2005) |
|  | KSE2 | I have the expertise needed to provide valuable knowledge to my institution |  |
|  | KSE3 | It really makes a difference when I add to the knowledge others are likely to share through the VCC online platform. |  |
|  | KSE4 | Other individuals in my institution cannot provide more valuable knowledge than I can. |  |
| Commitment | COM1 | I am willing to put in a great deal of effort in order to help my institution to be successful | (Bozeman and Perrewé, 2001) |
|  | COM2 | I really care about the fate of my institution |  |
|  | COM3 | I feel sense of pride being a part of my institution |  |
|  | COM4 | I feel loyal towards my institution |  |

| Factor | Item Code | Adopted items | Source of Items |
| --- | --- | --- | --- |
|  |  |  |  |
| Web Engagement | WEG1 | The university VCC online platform holds my attention | (Webster and Ahuja, 2006) |
|  | WEG2 | The university VCC online platform excites my curiosity |  |
|  | WEG3 | The university VCC online platform stimulates my imagination |  |
|  | WEG4 | The university VCC online platform is intrinsically interesting |  |
| Interactional Engagement | ING1 | I like to post ideas on my university VCC online platforms | (So.Kevin *et al.*, 2014) |
|  | ING2 | I enjoy exchanging ideas with others through my university VCC platforms. |  |
|  | ING3 | I often participate in my university’s VCC activities |  |
|  | ING4 | I like to get involved in sharing ideas discussions via university VCC platforms |  |

Items related to engagement factors

| Factor | Item Code | Adopted items | Source of Items |
| --- | --- | --- | --- |
|  |  |  |  |
| Learning benefits | LBN1 | The university’s VCC platform enhances my knowledge about the product\service in my institution | (Nambisan and Baron, 2009)  Learning: (three-item scale based on customer interviews; Franke and Shah, 2003; Hertel *et al*., 2003; Wasko and Faraj, 2000) |
|  | LBN2 | The university’s VCC platform helps me to obtain solutions to specific service related problems |  |
|  | LBN3 | The university’s VCC platform enhances my knowledge of the institution’s advances in related products, services and technology. |  |
| Social benefits | IMG1 | Sharing my ideas through VCC online platform improves my reputation in the university. | (Kankanhalli *et al.*, 2005) |
|  | IMG2 | Sharing my ideas through VCC online platform improves others’ recognition of me. |  |
|  | IMG3 | When I contribute my ideas through VCC online platform, the people I work with respect me. |  |
|  | IMG4 | When I contribute my ideas through VCC online platform, my superiors praise me |  |
|  | IMG5 | People in my university who contribute their ideas through VCC online platform have more prestige than those who do not |  |
| Intrinsic  Benefits | INB1 | I feel happy when my ideas help to solve complex problems | (Yuan and Woodman, 2010) |
|  | INB2 | I feel satisfied when my ideas improve a service or product |  |
|  | INB3 | I feel accomplished when I engage in creative thinking |  |
|  | INB4 | I feel pleased at creating new procedures for work tasks |  |
| Extrinsic  Benefits | EXB1 | My university gives monetary rewards in return for good contributed ideas | (Bock *et al.*, 2005) |
|  | EXB2 | My university gives bonus points for promotion in return for good shared ideas |  |
|  | EXB3 | My university gives non-monetary rewards (such as (certificates, gifts …etc.) in return for good contributed ideas |  |

Items related to **Perceived benefits** factors
